# Patient-derived ovarian cancer organoids capture the genomic profiles of primary tumours applicable for drug sensitivity and resistance testing

**DOI:** 10.1038/s41598-020-69488-9

**Published:** 2020-07-28

**Authors:** Yoshiko Nanki, Tatsuyuki Chiyoda, Akira Hirasawa, Aki Ookubo, Manabu Itoh, Masaru Ueno, Tomoko Akahane, Kaori Kameyama, Wataru Yamagami, Fumio Kataoka, Daisuke Aoki

**Affiliations:** 1 Department of Obstetrics and Gynecology, Keio University School of Medicine, Tokyo, Japan; 2 JSR-Keio University Medical and Chemical Innovation Center (JKiC), Keio University School of Medicine, Tokyo, Japan; 3 Department of Clinical Genomic Medicine, Graduate School of Medicine, Dentistry and Pharmaceutical Sciences, Okayama University, Okayama, Japan; 4 JSR-Keio University Medical and Chemical Innovation Center (JKiC), JSR Corp., Tokyo, Japan; 5 Genomics Unit, Keio Cancer Center, Keio University School of Medicine, Tokyo, Japan; 6 Department of Pathology, Keio University School of Medicine, Tokyo, Japan; 7 Present Address: Department of Pathology, Showa University Northern Yokohama Hospital, Yokohama, Japan; 8 Present Address: Department of Obstetrics and Gynecology, International University of Health and Welfare School of Medicine, Narita, Japan

**Keywords:** Gynaecological cancer, Ovarian cancer, Cancer models

## Abstract

The use of primary patient-derived organoids for drug sensitivity and resistance testing could play an important role in precision cancer medicine. We developed expandable ovarian cancer organoids in < 3 weeks; these organoids captured the characteristics of histological cancer subtypes and replicated the mutational landscape of the primary tumours. Seven pairs of organoids (3 high-grade serous, 1 clear cell, 3 endometrioid) and original tumours shared 59.5% (36.1–73.1%) of the variants identified. Copy number variations were also similar among organoids and primary tumours. The organoid that harboured the *BRCA1* pathogenic variant (p.L63*) showed a higher sensitivity to PARP inhibitor, olaparib, as well as to platinum drugs compared to the other organoids, whereas an organoid derived from clear cell ovarian cancer was resistant to conventional drugs for ovarian cancer, namely platinum drugs, paclitaxel, and olaparib. The overall success rate of primary organoid culture, including those of various histological subtypes, was 80% (28/35). Our data show that patient-derived organoids are suitable physiological ex vivo cancer models that can be used to screen effective personalised ovarian cancer drugs.

## Introduction

Patient-derived tumour organoids have become important preclinical model systems in both cancer research and clinical settings^1^. In contrast to patient-derived xenograft (PDX) mouse models that need a large amount of surgical specimen and 4–8 months for development^2^, organoids can be cultured from patient materials and can be expanded with high efficiency in a relatively short period (typically < 1 month). Organoids from mouse intestine, as well as from various other mouse and human tissues, including the colon, stomach, liver, lung, prostate, and pancreas, have been established^3,4^. Patient-derived tumour organoids have also been generated from the colon, pancreas, prostate, breast, gastric, lung, oesophageal, bladder, ovarian, kidney, and liver tumour tissues^1^. Organoids maintain the key genetic and phenotypic features of primary tumours, thereby, enabling their use in a broad range of applications, such drug development and identification of the best therapeutic regimen for each patient.

Ovarian cancer is a devastating disease, with 295,000 new patients and 185,000 deaths each year, worldwide^5^. The relative 5-year survival rate is 47% and has not apparently increased in the last 40 years. Debulking surgery with platinum-combination chemotherapy is usually administered to patients, irrespective of the histological subtypes, namely high-grade serous (HGSC), endometrioid (EM), clear cell (CCC), and mucinous carcinoma. HGSC comprises 70–80% of all ovarian cancer cases and is characterised by *TP53* mutation, with many chromosomal aberrations^6^. EM and CCC are endometriosis-associated ovarian cancers that frequently harbour the *ARID1A* mutation. Mucinous ovarian cancer is a rare tumour that accounts for 3% of all ovarian cancers and harbours *KRAS* mutation, *ERBB2* amplification, or *TP53* mutation^7^. Anti-VEGF antibody, bevacizumab, and PARP inhibitors, olaparib, rucaparib, and niraparib, are the molecular targeted drugs used in the clinical treatment of ovarian cancer. PARP inhibitors are effective against tumours with homologous recombination deficiency (HRD) and are mainly used against HGSC. Mismatch repair-deficient tumours make up less than 2% of the epithelial ovarian cancer^8^, and the overall response rate of single-agent immune checkpoint blockade by pembrolizumab was reported to be 4.1% in ovarian cancer^9^. Therefore, there are still emergent needs in ovarian cancer treatment for novel molecular targeted drugs and biomarkers for selecting the most effective therapeutic regimens.

Recently, established ovarian cancer organoids that capture the genomic features of primary tumours have been reported^10–12^. Here, we have effectively established ovarian cancer organoids from several histologic subtypes of ovarian cancer that can be utilised for biomedical applications, including drug sensitivity and resistance testing (DSRT).

## Results

### Establishment of primary ovarian cancer organoids

**Figure 1:**
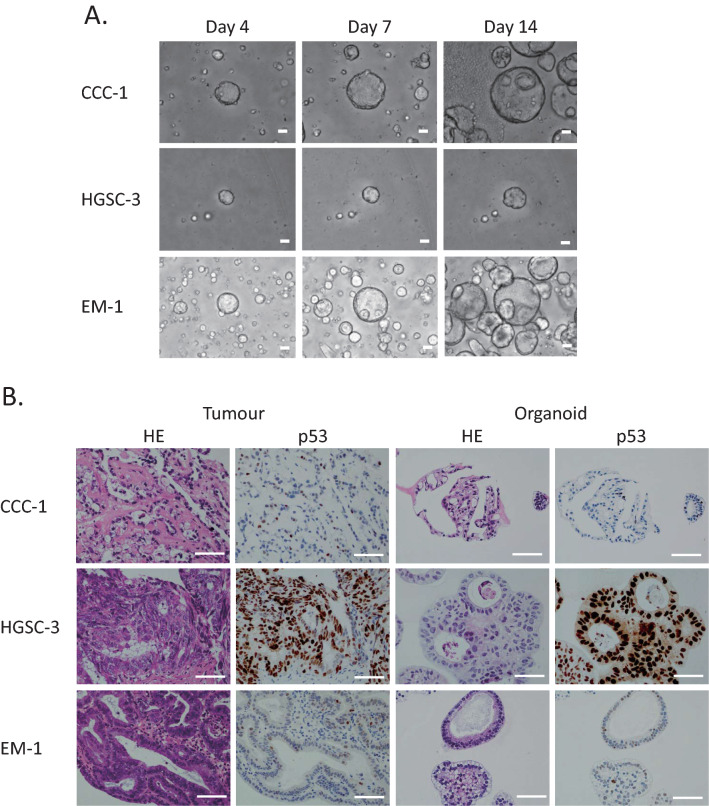
Patient-derived primary ovarian cancer organoids maintain the histological architecture and p53 positivity of the tumour subtype from which they are derived. (**A**) Brightfield microscopy images of the organoid lines. Scale bars 100 µm. (**B**) Haematoxylin and eosin (H&E) staining and p53 immunohistochemistry of primary ovarian tumours and derived organoids. It is noteworthy that organoids recapture the histologic features of the primary tumours (clear cell ovarian cancer, high-grade serous ovarian cancer, and endometrioid ovarian cancer) and p53 staining pattern. Scale bar = 100 µm.

First, we established the protocol to culture and expand single cells dissociated from primary ovarian cancers. Culturing dissociated single tumour cells in Matrigel with a cocktail medium of niche factors (WNT-3A, R-Spondin, etc.) enabled us to develop ovarian cancer organoids from different histologic subtypes (HGSC, EM, CCC) of stage I–III ovarian cancer patients within 1–3 weeks (Fig. 1A, Table 1). We studied the growth of ovarian cancer organoids using over 20 combinations of various niche factor culture cocktails. Here, we present the organoids cultured with the cocktail medium most effective for multi-tissue type culture. The overall success rate of the primary organoid culture was 80% (28/35) (Table 2). The established organoids captured the histological characteristics and p53 positivity of the primary tumours (Fig. 1B).

**Table 1:** Characteristics of organoid cases.

Case	Age at diagnosis	Stage	NAC	Debulking status	Observation period	Recurrence	Time to recurrence after platinum therapy	Status
HGSC-1	47	IIIC	None	Optimal	26 M	Yes	18 M	Alive with disease
HGSC-2	74	IIIC	ddTC + Bev	Optimal	26 M	Yes	9 M	Alive with disease
HGSC-3	77	IA	None	Complete	25 M	No	NA	NED
CCC-1	50	IA	None	Complete	26 M	No	NA	NED
EM-1	46	IC1	None	Complete	22 M	No	NA	NED
EM-2	42	IC2	None	Complete	23 M	No	NA	NED
EM-3	41	IIIB	None	Complete	22 M	No	NA	NED

*NAC* neoadjuvant chemotherapy, *ddTC* dose-dense paclitaxel carboplatin, *Bev* bevacizumab, *M* month, *NED* no evidence of disease, *NA* not applicable.

**Table 2:** Success rate of organoid culture and derived organoid lines from each histologic subtype of ovarian tumour.

	Number of cases	Number of successful primary organoid culture	Success rate of primary organoid culture (%)	Number of derived organoid lines
HGSC	10	9	90	3
EM	5	3	60	3
CCC	10	10	100	9
MC	0	0	0	0
MBT	3	3	100	2
Others	7	3	43	1
Total	35	28	80	18

*HGSC* high-grade serous, *EM* endometrioid, *CCC* clear cell, *MC* mucinous, *MBT* mucinous borderline tumour

Others include dysgerminoma, thecoma, serous cystadenofibroma, carcinosarcoma, and fibroma. Organoid line was defined as an organoid that could be made from a single cell culture and for which a serial passage was successful for four times.

### Capture of primary tumour genomic characteristics by organoids

**Figure 2:**
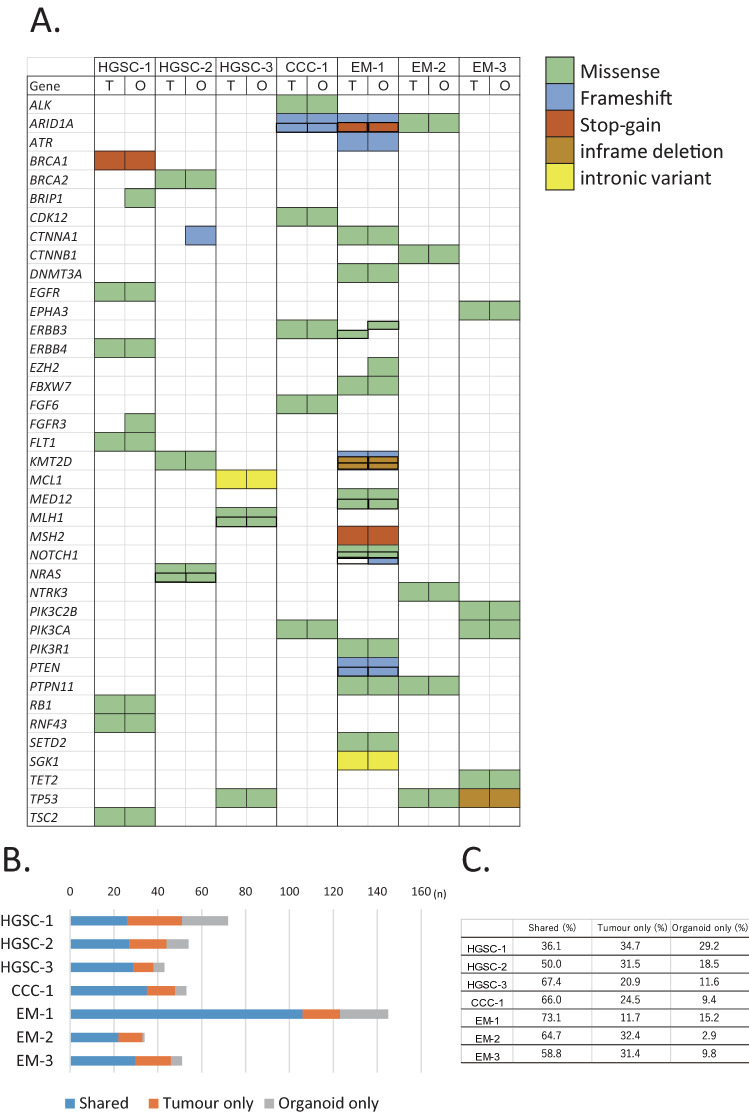
Organoids preserve the genetic alterations of the original tumour. (**A**) A cancer-related set of variants found in organoids and primary tumours (full list is shown in Supplementary Table 1). The type of mutation is indicated in the legend. Corresponding gene variant of tumour and organoid side by side in a same patient indicates a same variant. *T* tumour, *O* organoid. (**B**) The stacked bar graphs showing the total number of mutations per patient sample identified in both tumour and derived organoid, tumour only, and organoid only. (**C**) The percentage of shared, tumour only, and organoid only variants are indicated. Primary tumours and organoids share 59.5% (36.1–73.1%) of the variants.

**Figure 3:**
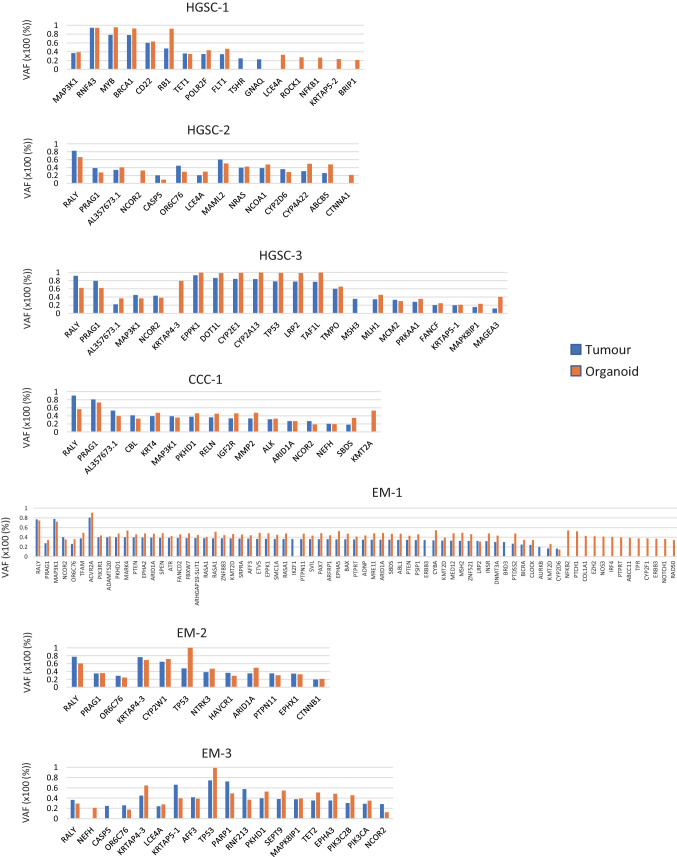
Prevalence of subclonal populations as revealed by the examination of variant allele frequency (VAF). Genes with a VAF of 40–60% identified both in tumour and organoid were excluded from the graph as these may be germline variants. Genes with a VAF of < 20% both in tumour and organoid were also excluded.

To compare the genomic characteristics of the parental tumours and derived organoids, we performed targeted capture sequencing of 1,053 cancer-related genes in the seven pairs (3 HGSC, 1 CCC, 3 EM) of organoids and primary tumours. The median passage number of organoids for analysis was 4 (range: 2–5). The analysis revealed that the pairs shared pivotal DNA variants, such as *BRCA1*, *BRCA2*, *MLH1*, *PIK3CA,* and *TP53* (Fig. 2A, Supplementary Table 1). HGSC-1 harboured a stop-gain mutation in *BRCA1* (p.L63*, pathogenic); CCC-1 had a frameshift mutation in *ARID1A* (p.P1995Lfs*22, p.Q1098Rfs*16); EM-1 had a frameshift mutation (p.K1072Nfs*21) and a stop-gain mutation (p.R1276*) in *ARID1A*; and EM-2 had a missense mutation in *ARID1A* (p.P251A) in both organoids and tumours. EM-1 also possessed a stop-gain mutation of the *MSH2* gene (p.G220*). In total, 59.5% (range: 36.1–73.1%) of the variants were shared among organoids and primary tumours. A total of 26.7% (range: 11.7–34.7%) of the variants were seen only in the tumour and 13.8% (range: 9.4–29.2%) were identified only in the organoid (Fig. 2B,C). EM-1 displayed a hypermutation pattern (145 gene variants). The variant allele frequency (VAF) was similar among organoids and primary tumours (Fig. 3, Supplementary Table 1). The VAF of 77.8% for *BRCA1* variant (p.L63*) seen in the HGSC-1 tumour indicates a loss of heterozygosity (LOH) in the tumour or the presence of uniparental disomy with a germline mutation. The VAF of 92.4% for *BRCA1* p.L63* in the organoid indicates that epithelial cells were concentrated in the organoids. Following the results of *BRCA1* variant analysis in the organoids, we performed genetic counselling of the patient. Genetic test revealed that the patient had a germline *BRCA1* variant. The VAF of *RB1* variant (p.E672Q) was 47.3% in HGSC-1 tumour and 92.2% in HGSC-1 organoid, which indicates that the *RB1* wild type allele was lost during organoid development. HGSC-3 organoid and parental tumour both showed a ClinVar pathogenic *TP53* variant (p.R248Q) with LOH. During development of the HGSC-3 organoid, a *KRTAP4-3* variant was acquired with LOH in the main clone. In CCC-1, the VAF of *ARID1A* was 26.8% in the tumour and 27% in the organoid, which indicates that a subclone was maintained in the organoid culture. EM-1 displayed a hypermutation pattern, most of which was maintained in the organoid. EM-2 organoid gained a LOH of the *TP53* variant (p.L130V), whereas the wild type allele of *TP53* was identified in the tumour.

### Copy number variations of tumours and organoids

**Figure 4:**
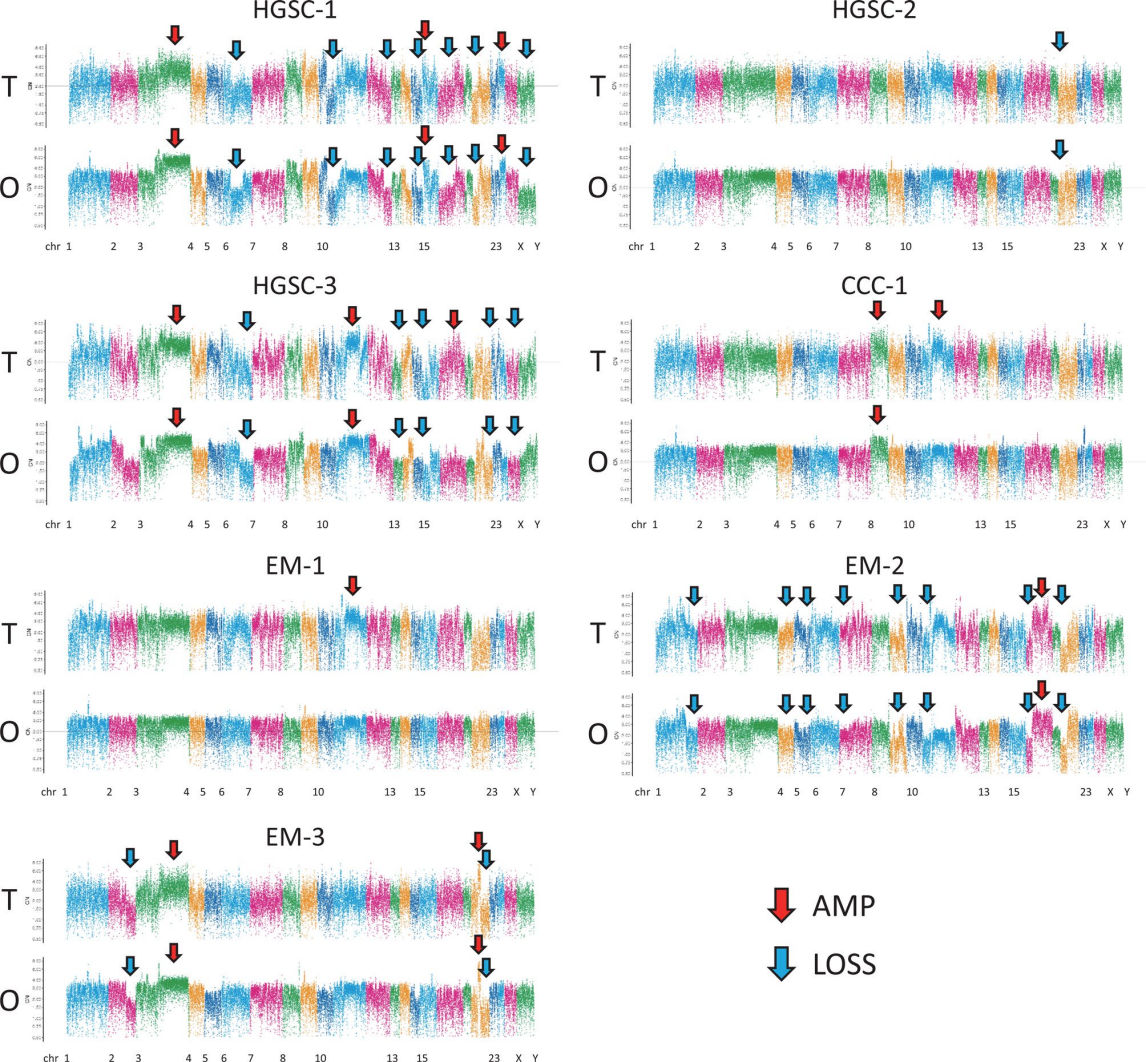
Copy number variation (CNV) profiles with correlations (Pearson's r) of tumour tissue and organoid samples in the seven cases. CNV profiles of the tumour tissue and organoids were created using a comprehensive capture-based cancer panel of 1053 genes. The pileup file was generated from processed BAM file that were used for variant call using SAMtools v. 1.2 (https://samtools.sourceforge.net/). The log-base-2 of the ratio of depth to average depth was calculated. CN was computed using the log-base-2 ratio and plotted. R script for these processes ran on R 3.4 (https://www.R-project.org). Red allow, chromosomal region with amplification; Blue allow, chromosomal region with loss. T: tumour, O: organoid.

Copy number variations (CNVs) of the seven pairs of ovarian organoids and primary tumours showed a similar pattern of amplifications and losses throughout the chromosomes (Fig. 4). HGSC-1 and HGSC-3 had many amplifications and losses that may reflect HRD (HRD-like). HGSC-2 showed scarce CNVs (non-HRD like). Chromosome 8 amplification was seen in both the parental tumours and organoids of CCC-1, whereas amplification in the region of chromosome 11 was only seen in the primary tumour. Most of the changes in the copy number of chromosomes in the tumours were inherited in the organoids, and the organoids did not acquire major novel chromosomal aberrations.

### Organoid usability for personalised DSRT

**Figure 5:**
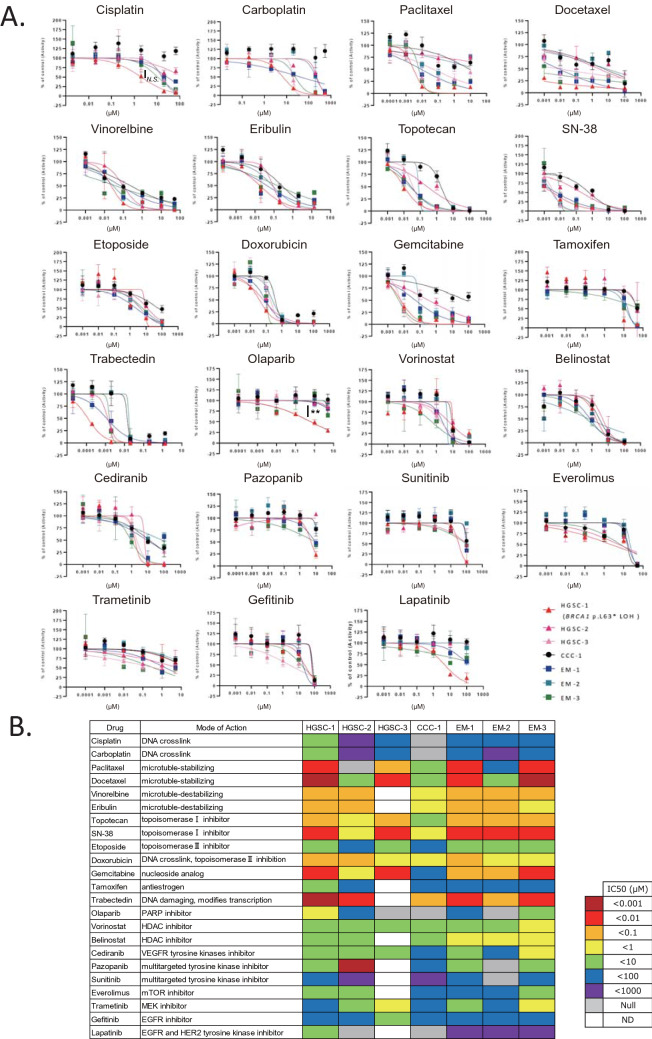
Ovarian cancer organoids as a platform for drug screening. (**A**) Dose–response curves of the organoid lines treated with 23 FDA-approved compounds. Dots represent the mean of the technical duplicates. Error bars represent the SEM of technical duplicates. ** < *p* = 0.01, *N.S.*: not significant (one-way ANOVA). Data analyses were performed using the GraphPad Prism 7.0b software. (**B**) Summary of the 23 FDA-approved compounds used in the drug sensitivity and resistance testing (DSRT) and the results. The corresponding colours for IC50 are depicted in the legend. HGSC-1 (*BRCA1* pathogenic variant) showed higher sensitivity to cisplatin and olaparib compared to others. CCC-1 showed resistance to commonly used drugs for ovarian cancer; paclitaxel, carboplatin, and olaparib compared to other organoids. N = 3 distinct organoid lines. *ND* not determined.

Finally, we performed DSRT using 23 FDA-approved drugs. HGSC-1 and HGSC-3 (HRD-like) displayed a similar drug sensitivity pattern that was different from that of HGSC-2 (non-HRD like) (Fig. 5A,B, Supplementary Table 2). HGSC-2 showed resistance to most of the drugs, except trabectedin. CCC-1 was resistant to platinum drugs (cisplatin and carboplatin) and paclitaxel, the key drugs in ovarian cancer therapeutics (Fig. 5A,B, Supplementary Table 2). HGSC-1 that harbours the deleterious *BRCA1* variant and loss of wild type allele showed higher sensitivity to the PARP inhibitor, olaparib (*p* < 0.01), compared to other organoids (Fig. 5A,B). HGSC-1 also showed a tendency of higher sensitivity to cisplatin, although it was not significant (*p* = 0.14) (Fig. 5A,B). HGSC-1 was also sensitive to paclitaxel, docetaxel, topotecan, SN-38, gemcitabine, and trabectedin. Time interval to recurrence after completion of first-line platinum regimen was 18 months in HGSC-1, which was longer than that in HGSC-2 (9 months), showing concordance with the results of DSRT (Table 1, Fig. 5A,B).

## Discussion

Ovarian cancer is a heterogeneous disease that needs an appropriate tumour model for the development of novel therapeutics. We have developed patient-derived ovarian cancer organoids that capture the in vivo architecture, genetic signature, and heterogeneity of tumours. In accordance with studies carried out on ovarian cancer and other tumours, such as those of the colon, breast, and bladder, our ovarian cancer organoids retained both the histological and genetic features and intra-tumoural heterogeneity of the original tumours^10,13–15^. Our method enables the establishment of organoids from several histological subtypes of ovarian cancer, efficiently from a single cell, within 3 weeks. The established organoids recapitulated the CNVs of primary tumours. An organoid with *BRCA1* variant (HGSC-1) displayed many chromosomal aberrations, which are characteristic of HGSC. A total of 59.1% of gene variants were shared among organoids and corresponding tumours in our study, which was lower than the percentage (98%) of shared variants reported previous^12^. The difference may be attributed to the fact that we performed DNA sequencing at median passage number of 4 (> 4 weeks) but in the previous study, it was done after a short period (7–10 days) of culture. The pivotal DNA variants for tumourigenesis were shared among organoids and tumours in our study and the shared variants were reported to be maintained even after prolonged culture^10^.

In the DSRT, organoid from clear cell ovarian cancer (CCC-1) showed resistance to paclitaxel, cisplatin, and carboplatin compared to the other organoids; this was consistent with the fact that clear cell ovarian cancer is resistant to platinum-based chemotherapy (response rate: clear cell 11.1%, serous 72.5%)^16^. CCC-1 has mutations in the SWItch/Sucrose Non-Fermentable (SWI/SNF) related genes, *PBRM1* (p.P1460L) and *ARID1A* (p.P1995Lfs*22, p.Q1098Rfs*16), indicating that immune checkpoint blockade might be an effective treatment strategy for this tumour^17,18^. HGSC-1 and HGSC-3 patients have HRD like CNVs, whereas HGSC-2 has limited CNVs (Fig. 4). HGSC-1 and HGSC-3 showed sensitivity towards paclitaxel treatment; however, HGSC-2 was resistant to paclitaxel (Fig. 5A,B, Supplementary Table 2). HGSC-1 harbours a pathogenic *BRCA1* variant and is sensitive to the PARP inhibitor, olaparib, and cisplatin compared to other organoids, which indicates that using organoid-based models is a reliable strategy for DSRT. In fact, HSGC1 and HGSC-2 were both from FIGO stage IIIC tumours. The disease-free period after platinum therapy was longer in HGSC-1 compared to that in HGSC-2, which can be considered as a reflection of DSRT (Table 1, Fig. 5A,B). Kopper et al. also reported that in vitro drug sensitivity was recapitulated in vivo using xenotransplantation of ovarian cancer organoids^10^.

As drug responses are more diverse and correlate better with genomic alterations in the 3D culture than in the 2D culture^19^, organoids can be considered as an appropriate culture format for drug sensitivity assays in translational research and precision medicine. PDX of ovarian cancer also recapitulated the diversity of genomic alterations in tumours and can be used for drug testing^20,21^; however, organoids are considered a better 3D culture system than PDX in terms of (1) amount of tumour needed, (2) engraftment time, and (3) engraftment success rate^2^.

The limitations of organoid models include the absence of cancer stroma, such as fibroblasts, blood vessels, and immune cells. Recently, however, the air–liquid interface method that retains tumour immune microenvironment was reported^22^. A PDX mouse model can be efficiently created via engraftment of organoids, which also enables the assessment of cancer stroma interactions. Organoid methodology allowing for cancer stroma integration is a vital next step in this field.

The difference between our method and a previously reported one^10^ is that we did not use heregulinβ-1, nicotinamide, forskolin, hydrocortisone, and estradiol, but used gastrin and insulin-like growth factor. Fibroblast growth factor, WNT, noggin, and R-spondin1 were the common niche factors. The primary organoid could be efficiently established with our method with a success rate of 80% (Table 2), which is similar to the success rate reported previously^11^.

In conclusion, the ovarian cancer organoids that we describe here recaptured the histological and genomic features of primary tumours, and thereby, present a useful platform for drug screening. By making it possible to progress from establishment to drug testing in a short time, this ovarian cancer organoid platform may serve as a pivotal experimental model, making it possible to predict drug responses before its administration to patients.

## Methods

### Sample collection and tissue processing

The collection of patient data and ovarian cancer tissues was performed at the Keio University Hospital with the approval of the institutional ethics committee (Approval No. 20070081). This study was performed in accordance with all relevant guidelines and regulations. All patients participating in this study signed informed consent forms. Ovarian cancer tissue was collected during surgery, and samples were stored in a sample storage solution at 4 °C during transportation to the laboratory. The sample storage solution consisted of Advanced DMEM/F12 (Thermo Fisher Scientific), 2 mM HEPES (Thermo Fisher Scientific), 1 × GlutaMAX-I (Thermo Fisher Scientific), and 200 U/mL penicillin/streptomycin (Thermo Fisher Scientific). The sample delivery time was 15–90 min. On arrival in the laboratory, the tissue samples were set on a sterile petri dish on crushed ice. Identifiable necrotic tissue and fat tissue were removed as much as possible and their size and weight were measured. An incision was made in the middle of the tissue to obtain a 5-mm slice for paraffin embedding of the primary tumour. Subsequently, the tumour was dissected to a 5 mm square under sterile conditions. A few pieces were stored at − 80 °C in 2 mL tubes with Recovery Cell culture freezing medium (Thermo Fisher Scientific) for later use. For organoid preparation, tissue samples were collected in 50 mL tubes containing HBSS 1 × (Thermo Fisher Scientific) and incubated on ice for 5 min. Thereafter, the supernatant was discarded. This wash step was repeated three times using HBSS 1 × . The cleaned tissue sample was placed on ice in a new petri dish, 100 µL of HBSS 1 × was added, and the sample was then minced into a paste. The minced tissue was collected in a new 50 mL tube and centrifuged at 300× *g* for 5 min at room temperature. The supernatant was discarded, pellet was gently loosened, and an enzymatic degradation solution containing HBSS 1 × , collagenase I (FUJIFILM Wako Pure Chemical Corporation), dispase II (FUJIFILM Wako Pure Chemical Corporation), Rock Inhibitor (FUJIFILM Wako Pure Chemical Corporation, CultureSure Y-27632), and DNase I (Roche) were added. The mixture was placed in a 50 mL tube in a water bath at 37 °C and shaken at 180–200 rpm for 30 min or up to 90 min. After the first 30 min, the dispersion status and live cells were checked every 15 min. The reaction was stopped when most of the cells had dispersed into single cells. The mixture was collected and dripped through a cell strainer on a new 50 mL tube to remove any residual tissue. The suspension was centrifuged at 300× *g* for 5 min at room temperature, the supernatant was removed, and the pellet was loosened. In case of a visible red pellet, erythrocytes were lysed in ACK Lysis buffer (Thermo Fisher Scientific) for 5–10 min at room temperature followed by two wash steps with 45 mL of HBSS 1X and centrifugation at 300× *g* for 5 min.

### Organoid culture and passaging

The concentration of the cell suspension was normalised to 1.0 × 10^4^ cells/drop and the suspension was centrifuged at 300× *g* for 5 min at room temperature. The cell pellet was then suspended in Matrigel (Corning), and 25 μL drops of matrix cell suspension were allowed to solidify on a pre-warmed 48-well plate at 37 °C for 15 min. We then calculated the volume to be transferred from the cell suspension into a new tube. On stabilisation of the Matrigel, we added the organoid medium cocktail. Advanced DMEM/F12 (Thermo Fisher Scientific) was supplemented with 2 mM HEPES (Thermo Fisher Scientific), 1 × GlutaMAX-I (Thermo Fisher Scientific), 1X B27 supplement (Thermo Fisher Scientific), 10 nM Leu15-Gastrin I (Sigma-Aldrich), 1 mM N-acetylcystein (Sigma-Aldrich), 100 ng/mL recombinant human IGF-1 (R&D Systems), 50 ng/mL recombinant human FGF-2 (PeproTech), 20% Afamin/Wnt3a CM (JSR Life Sciences), 1 µg/mL humanR-spondin (R&D Systems), 100 ng/mL Noggin (PeproTech), 500 nM A-83-01 (Tocris Bioscience), 200 U/mL penicillin/streptomycin (Thermo Fisher Scientific), and 10 µM Y-27632 (FUJIFILM Wako Pure Chemical Corporation). The medium was changed every 3–4 days, and the organoids were passaged at a 1:2–3 dilution every 1–4 weeks. Images of the organoids were taken every 3–4 days using a microscope. Organoids were mechanically and enzymatically dissociated into small clusters for passaging and collected in a 10 mL tube and sheared with a 1000 µL pipet tip without a filter. Thereafter, 100 µL of TrypLE Express (Invitrogen) was added and the mixture was incubated for 5 min at 37 °C. Subsequently, the organoids were centrifuged at 400× *g* for 3 min, washed with PBS, and centrifuged again briefly. All the seven organoids were successfully passaged and grown into organoids even after being isolated into single cells. For preservation of a stock, organoids in a Matrigel drop were suspended with Recovery Cell Culture Freezing Medium (Thermo Fisher Scientific), collected in a 2 mL tube, and then gradually frozen at − 80 °C using BICELL (Nihon Freezer).

### HE staining and immunohistochemistry of original tumours and organoids

Tissues and organoids were processed for paraffin sectioning using standard protocols. Matrigel embedded organoids were suspended in Cell Recovery Solution (Corning, 500 µL/well) and collected in a 15 mL tube. The organoid suspension was occasionally mixed with gentle pipetting for 30 min on ice to completely solubilise the Matrigel. The tube was then placed on ice to precipitate the organoids. The supernatant was removed, and organoids were washed with a small amount of cold PBS. Organoids were solidified using iPgell (NIPPON Genetics), and organoid blocks were fixed with 4% paraformaldehyde (PFA) for 20 min at room temperature, before embedding in paraffin. Fresh cancer tissue was embedded in paraffin after formalin fixation. After deparaffinisation, 5 μm sections were stained with haematoxylin–eosin (H&E) and tumour protein p53 (DO-7, Dako). The organoids and primary tumour sections were evaluated for morphological and immunostaining similarity by the pathologist.

### DNA targeted analysis of original tumour and organoids

DNA was isolated from 6–8 wells of cultured organoids with the QIAamp DNA Mini Kit (QIAGEN) according to the manufacturer’s instructions. Original tumour DNA was isolated from formalin-fixed, paraffin-embedded samples with the QIAamp DNA Mini Kit (QIAGEN). DNA purity and concentration were examined using the NanoDrop2000 spectrophotometer and Qubit 2.0 Fluorometer, respectively. The qualified genomic DNA sample was randomly fragmented with a Covaris ultrasonicator and adapters were ligated to both ends of the resulting fragments. The extracted DNA was then amplified via ligation-mediated PCR (LM-PCR), purified, and hybridised to the Roche NimbleGen SeqCap EZ Exome probe. Non-hybridised fragments were then washed off. Both the non-captured and captured LM-PCR products were subjected to quantitative PCR to estimate the magnitude of enrichment. Target enrichment was performed with a cancer panel that targeted 1,053 cancer-related genes (Beijing Genomics Institute). Each captured library was then loaded on an Illumina Hiseq sequencing platform, and high-throughput sequencing was performed independently for each captured library to ensure that each sample met the desired average fold-coverage. Raw image files were processed with Illumina base calling Software 1.7 with default parameters and the sequences of each individual were generated as 90/100 bp paired-end reads^23,24^.

### Genomic analysis

After removing the adaptor reads, the clean reads were mapped to the reference genome (hg19) using Burrows–Wheeler Alignment with maximal exact matches (BWA-MEM), v. 0.7.12^25^. Read mapping was followed by indel-realignment by the assembly based realigner (ABRA), v. 0.97^26^. Duplicate reads were marked for removal. SNVs and indels were called using VarScan, v. 2.4.2^27^. The functional effect of the SNVs and indels were predicted using SnpEff v. 4.2^28^. CNV was detected using an in-house algorithm of Mitsubishi Space Software. To determine the clinical actionability, all the variants were mapped to three clinical annotation databases, ClinVar (downloaded on 2017–02)^29^, COSMIC v. 81^30^, and CIViC (downloaded on 2016–02)^31^. Common variants were detected using the following criteria: allele frequency of more than 0.01 in any of the public database, Exome Aggregation Consortium database (ExAC) (https://exac.broa-dinstitute.org/)^32^, Human Genetic Variation Database (HGVD) (https://www.genome.med.kyoto-u.ac.jp/)^33^, and Tohoku Medical Megabank Organization 2KJPN data (ToMMo 2KJPN) (https://jmorp.megabank.tohoku.ac.jp/)^34^. HGVD and ToMMo 2KJPN were used as a reference for the Japanese controls.

### DSRT on organoids

Organoids were collected 4–5 days after passage and filtered through a 100 μm cell strainer to remove any large clumps. Values for each drug concentration point were averages of the values for the triplicate wells. Drugs were added 2 days after embedding. We selected 23 FDA-approved drugs including those covered by health insurance in Japan for ovarian cancer and endometrial cancer. Depending on the properties of the individual drugs, the concentrations ranged from 10 μmol/L to 128 pmol/L or from 100 μmol/L to 1.28 nmol. Cell viability was assayed using CellTiter-Glo 3D (Promega) on day 6. Data analyses were performed using the GraphPad Prism 7.0b software to calculate IC_50_^23^.

## Data availability

The datasets generated during and/or analysed during the current study are available from the corresponding author on reasonable request.

## Supplementary information

Supplementary Table legends.

Supplementary Table 1.

Supplementary Table 2.
