# Supplementary material for: Patient-derived ovarian cancer organoids capture the genomic profiles of primary tumours applicable for drug sensitivity and resistance testing
Source: Sci Rep. 2020 Jul 28;10:12581. doi: 10.1038/s41598-020-69488-9 (PMC7387538; doi:10.1038/s41598-020-69488-9)
Supplement: Supplementary file 1 — Supplementary Table legends. [file 41598_2020_69488_MOESM1_ESM.docx]

# Patient-derived ovarian cancer organoids capture the genomic profiles of primary tumours applicable for drug sensitivity and resistance testing

Yoshiko Nanki^1, 2^, Tatsuyuki Chiyoda^1, 2^*, Akira Hirasawa^1, 2, 3^*, Aki Ookubo^4^, Manabu Itoh^4^, Masaru Ueno^4^, Tomoko Akahane^2, 5^, Kaori Kameyama^6^, Wataru Yamagami^1^, Fumio Kataoka^1^, & Daisuke Aoki^1^

^1^Department of Obstetrics and Gynecology, Keio University School of Medicine, Tokyo. ^2^JSR-Keio University Medical and Chemical Innovation Center (JKiC), Keio University School of Medicine, Tokyo. ^3^Department of Clinical Genomic Medicine, Graduate School of Medicine, Dentistry and Pharmaceutical Sciences, Okayama University, Okayama. ^4^JSR-Keio University Medical and Chemical Innovation Center (JKiC), JSR Corp., Tokyo. ^5^Genomics Unit, Keio Cancer Center, Keio University School of Medicine, Tokyo. ^6^Department of Pathology, Keio University School of Medicine, Tokyo.

Supplementary Table 1. Full list of variants found in the organoids and the primary tumours. Variant allele frequency (×100 (%)) is indicated.

Supplementary Table 2. Summary of IC50 in the drug sensitivity and resistance testing (DSRT) of organoids using the 23 FDA-approved compounds. ND, not determined.
