# Supplementary material for: Patient-derived ovarian cancer organoids capture the genomic profiles of primary tumours applicable for drug sensitivity and resistance testing
Source: Sci Rep. 2020 Jul 28;10:12581. doi: 10.1038/s41598-020-69488-9 (PMC7387538; doi:10.1038/s41598-020-69488-9)
Supplement: Supplementary file 3 — Supplementary Table 2. [file 41598_2020_69488_MOESM3_ESM.docx]

|  | HGSC-1　（μM） | HGSC-2 （μM） | HGSC-3 （μM） | CCC-1 （μM） | EM-1 （μM） | EM-2 （μM） | EM-3 （μM） |
| --- | --- | --- | --- | --- | --- | --- | --- |
| Cisplatin | 2.0 | 135.1 | 17.7 | Null | 28.2 | 16.2 | 10.7 |
| Carboplatin | 6.7 | 229.8 | ~ 18.8 | Null | 40.8 | 207.2 | 24.2 |
| Paclitaxel | 0.003 | Null | 0.05 | 8.8 | 0.009 | 29.6 | 0.006 |
| Docetaxel | 2.7E-08 | 2.6 | 0.008 | 5.2 | 0.008 | 7.7 | 1.1E-05 |
| Vinorelbine | 0.02 | 0.1 | ND | 0.25 | 0.07 | 0.03 | 0.04 |
| Eribulin | 0.02 | 0.09 | ND | 0.26 | 0.04 | 0.08 | 0.15 |
| Topotecan | 0.01 | 0.27 | 0.04 | 1.8 | 0.03 | ~ 0.08 | 0.02 |
| SN-38 | 0.003 | 0.20 | 0.004 | 0.60 | 0.006 | 0.003 | ~ 0.01 |
| Etoposide | ~ 8.5 | 11.9 | 4.4 | 56.2 | 2.3 | 4.2 | 6.0 |
| Doxorubicin | 0.04 | 0.07 | 0.19 | 0.37 | 0.09 | 0.24 | ~ 0.11 |
| Gemcitabine | 0.006 | 0.34 | 0.006 | 67.8 | 0.05 | ~ 0.08 | 0.005 |
| Tamoxifen | ~ 9.8 | 47.3 | ND | 51.0 | 15.7 | 12.5 | 66.4 |
| Trabectedin | 0.0001 | 0.0013 | ND | ~ 0.014 | 0.0012 | ~ 0.017 | ~ 0.0017 |
| Olaparib | 0.63 | 24.2 | Null | Null | 23.3 | Null | ~ 5.3 |
| Vorinostat | ~ 9.7 | 6.5 | 2.1 | ~ 8.9 | 2.4 | ~ 4.4 | 0.60 |
| Belinostat | 1.8 | 4.5 | ND | 2.8 | 0.7 | 0.59 | 0.44 |
| Cediranib | 1.6 | ~ 6.2 | 2.1 | 12.4 | 8.9 | 75.0 | 0.9 |
| Pazopanib | 4.9 | 1.3E-06 | ND | ~ 12.8 | 9.3 | Null | 9.6 |
| Sunitinib | 31.4 | 106.7 | ND | ~ 104.8 | ~ 90.9 | Null | 92.0 |
| Everolimus | 2.1 | 5.4 | ND | 15.1 | 16.6 | 21.3 | 6.5 |
| Trametinib | 17.3 | 7.0 | 0.4 | 16.6 | 1.6 | 26.5 | 0.4 |
| Gefitinib | 23.4 | ~ 65.3 | 2.5 | ~ 74.6 | 18.7 | ~ 68.5 | 14.6 |
| Lapatinib | 4.6 | Null | ND | Null | 139.2 | ~ 132.7 | 635 |

Supplementary Table 2. Summary of IC50 in the drug sensitivity and resistance testing (DSRT) of organoids using the 23 FDA-approved compounds. ND, not determined.
